# The Exercise, Arterial Modulation and Nutrition in Youth South Africa Study (ExAMIN Youth SA)

**DOI:** 10.3389/fped.2020.00212

**Published:** 2020-04-29

**Authors:** Ruan Kruger, Makama Andries Monyeki, Aletta Elisabeth Schutte, Wayne Smith, Catharina Martha Cornelia Mels, Herculina Salomé Kruger, Anita Elizabeth Pienaar, Lebo Francina Gafane-Matemane, Yolandi Breet, Leandi Lammertyn, Gontse Gratitude Mokwatsi, Ankebé Kruger, Elmari Deacon, Henner Hanssen

**Affiliations:** ^1^Hypertension in Africa Research Team (HART); North-West University, Potchefstroom, South Africa; ^2^MRC Research Unit for Hypertension and Cardiovascular Disease, North-West University, Potchefstroom, South Africa; ^3^Physical Activity, Sport and Recreation Research Focus Area; North-West University, Potchefstroom, South Africa; ^4^School of Public Health and Community Medicine, University of New South Wales and The George Institute for Global Health, Sydney, NSW, Australia; ^5^Centre of Excellence for Nutrition, North-West University, Potchefstroom, South Africa; ^6^Optentia Research Focus Area, North-West University, Potchefstroom, South Africa; ^7^Department of Sport, Exercise and Health, University of Basel, Basel, Switzerland

**Keywords:** child health, blood pressure, obesity, arterial stiffness, retinal vessel diameters, nutrition, physical activity, psychosocial behavior

## Abstract

**Background:** The impact of a sedentary and unhealthy lifestyle on cardiovascular health is well-documented, however the current obesity and hypertension trends among children is concerning. The ExAMIN Youth SA study aims to investigate the impact of lifestyle behaviors (physical fitness/activity, dietary intake and psychosocial factors) involved in early vascular aging among South African children.

**Methods:** This study is an analytical, multidisciplinary, observational cohort study in a school-based setting. We aim to phenotype a cohort of ~1,000 primary school children (black and white boys and girls between ages 5–9 years) based on current clinical childhood conditions including hypertension and obesity. The primary phenotype is large artery stiffness and retinal microvascular diameters, both biomarkers of early vascular aging. The risk factors and mediators of early vascular aging and also responsible for the clinical conditions include physical inactivity, unhealthy diet, and life stress. Additionally, urinalysis and salivary analyses will be performed to identify biomarkers related to the pathophysiology of early vascular aging.

**Discussion:** In line with the growing prevalence of obesity and hypertension responsible for the development of early vascular aging from childhood to adulthood, this study will address the critical areas in which we observe unfavorable arterial modulation related to dietary behaviors, physical inactivity, and early life stress. Implementation of novel biological markers may further contribute to our understanding of early cardiovascular adaptations in childhood, and aid in the development of primary prevention programs.

**Trial registration:** The study was retrospectively registered on ClinicalTrials.gov on 15 August 2019 (NCT04056377).

## Background

Two of the most important future health care challenges include the global rise of hypertension and obesity. Elevated blood pressure and adiposity in childhood tracks into later life, and contribute to the burden of cardiovascular disease and type 2 diabetes (1, 2). The *Exercise, Arterial Modulation and Nutrition in Youth South Africa* (ExAMIN Youth SA) study was designed as a multidisciplinary cohort analytical study to increase our understanding of the complex mechanisms involved in the etiology of early cardiovascular alterations in a cohort of children (aged 5–9 years). This study will contribute to our understanding of the early pathophysiological changes related to unfavorable arterial modulation in childhood.

In recent years, high blood pressure and the importance of recognizing its harmful implications in children became momentous (3). However, there remains a lack of primary prevention implementation in childhood, where most impact can be made to curb early non-communicable disease onset. Many cardiovascular related complications have no early detectable clinical signs and symptoms, and may therefore go unnoticed. Hence, the potential ambivalence of not recognizing childhood hypertension (4). Elevated blood pressure and obesity are two of the modern day's most prominent risk factors for cardiovascular morbidity and mortality (5). These risk factors are becoming an even larger threat to child health, due to an increasing prevalence of unhealthy and sedentary lifestyle practices among children globally (6, 7). Worldwide analyses have indicated that the highest blood pressures are recorded in black populations (8). The vulnerable cardiovascular profile of black individuals is believed to result from a combination of factors such as rapid urbanization, physical inactivity, suboptimal dietary practices, and consequently, weight gain with an increased prevalence of childhood obesity (9).

The importance of the ExAMIN Youth SA study is the early detection of physical inactivity, unhealthy dietary behaviors and psychosocial stressors as underlying contributors of pre-clinical large- and small artery pathology—*arterial modulation*. In a previous study, we observed a high proportion (12.5%) of elevated blood pressure in black boys (aged 6–8 years), with higher arterial stiffness in all sections of the arterial tree, higher diastolic blood pressure and mean arterial pressure, a thicker carotid intima–media wall, increased total peripheral resistance and higher advanced glycosylation end-products compared to white boys of similar ages (10). Other studies also described high total peripheral resistance (11) and low socioeconomic status (12) to contribute to the burden of childhood hypertension especially among the black population. This phenotype underlined the increasing trend of early-onset vascular aging among black adult populations in South Africa.

By integrating our previous knowledge from the Arterial Stiffness in Offspring Study (also performed in children aged 6–8 years), we will further introduce urinary and salivary biomarkers that could aid in identifying early risk markers with the ability to predict potential future cardiovascular compromise due to interactions of lifestyle behaviors on arterial modulation. Among these risk markers are amino acids, acylcarnitines, and organic acids determined by novel technologies to identify metabolomic patterns known to be involved in adverse dietary behavior (13). Additionally, preliminary analyses in the Arterial Stiffness in Offspring Study yielded information of lower nitric oxide bioavailability in black compared to white boys (14), suggesting potential early signs of endothelial dysfunction. Therefore, in a larger sample in the ExAMIN Youth SA study, we aim to determine markers related to nitric oxide bioavailability such as urinary nitrite, nitrate, symmetric, and asymmetric dimethylarginines, dimethylamine, and malondialdehyde (15, 16).

In addition, we will assess the retinal vasculature in children as one of the primary outcomes. Retinal vessel diameters are valid microvascular biomarkers shown to be associated with a higher risk of hypertension and obesity, incidence of stroke and myocardial infarction as well as higher cardiovascular mortality in adults (17, 18). With elevated blood pressure not being benign in children and previously linked to target organ damage including increased carotid intima–media thickness (19) and left ventricular mass (20), this study will additionally explore the links with the retinal microvasculature as target organ damage marker in South African children. A previous study indicated that childhood obesity, high blood pressure, and physical inactivity were associated with retinal microvascular abnormalities, suggesting retinal vessel imaging as promising biomarkers for early risk stratification in children (21). No such study to date has examined retinal vessel diameters in South African children and analyzed associations with blood pressure, body composition, physical activity, and dietary or lifestyle factors. Moreover, concomitant assessment of large artery stiffness and retinal microvascular diameters allows for investigation of the cross-talk between the macro- and microcirculation. In a collaborative effort, this ExAMIN Youth SA study is aligned with the Exercise and Arterial Modulation in Children study from Basel, Switzerland (22), to allow cross-comparisons among two international cohorts, ethnicities and sex. Studies suggested that increased estrone levels in men are indicative of future diabetes risk (23) and associated with adverse lipid profiles and is apparent early in life prior to cardiovascular disease manifestations (24). However, studies in children are lacking in this regard. To establish the potential early differences and involvement of sex hormones in arterial modulation, estrone (one of the three most prominent estrogens) derived from peripheral tissue conversion in prepubertal children is of interest to investigate, especially due to its role in vascular reactivity and function as an anti-atherosclerotic agent (25).

Besides the abovementioned factors, psychosocial stress is considered to play a significant role in the development of cardiovascular disease (26). Psychosocial stress may have an impact on an individual's psychobiological processes (hypothalamic-pituitary-adrenal function, sympathetic nervous system function, endothelial dysfunction, inflammation, and autonomic control). Furthermore, researchers hypothesized that psychosocial stress might have a negative impact on health behaviors such as decreased motivation to be physically active (27). Stressful life events were found to have a negative association with self-reported health among children as well as older individuals who exercised infrequently (28). The majority of previous studies indicated that regular physical activity and improved aerobic functioning can potentially protect an individual against health risks associated with a higher perception of psychosocial stress (29). The hypothalamic-pituitary adrenal axis responses can be studied as indexed by cortisol reactivity to stressful experiences (30). The ExAMIN Youth SA study will employ a physical activity intervention in order to determine the beneficial effects of physical activity on HPA axis reactivity. Poor health and performance related fitness at a young age can negatively impact physical activity behaviors which can in turn influence cardiovascular health (31). Nevertheless, previous studies focused mostly on older children and adolescents while research investigating younger children (<8 years) is still lacking. By studying baseline cortisol levels and identifying those with higher hypothalamic-pituitary adrenal axis responses may aid in the implementation of future prevention strategies to curb early onset distress known to impact on cardiovascular health (32).

This study will implement state-of-the-art techniques and biochemical technologies to address especially behavioral contributors to hypertension and obesity development; and to subsequently provide a backdrop for school-based primary prevention interventions in South Africa.

## Methods

This study protocol followed the Standard Protocol Items: Recommendations for Interventional Trials guidelines (33). This protocol paper reports on the baseline and proposed follow-up procedures of the ExAMIN Youth SA study.

### Aim

The aim of this paper is to provide the protocol of the ExAMIN Youth SA study. The ExAMIN Youth SA study aims to determine the prevalence of childhood hypertension and obesity as clinical conditions in South Africa, with large artery stiffness and retinal microvascular diameters as primary contributors. We further aim to investigate the risk factors and mediators of both early vascular aging (arterial stiffness and retinal microvasculature) and the clinical conditions including physical inactivity, unhealthy diet, life stress and biomarkers.

### Outcomes

The study has the following outcomes based on primary school children in the North West province of South Africa:

1. To evaluate obesity and hypertension prevalence along with its determinants motor skills and physical fitness/activity, intake from food groups, and psychosocial behaviors.
2. To assess the association of body mass index and blood pressure with central pulse wave velocity and retinal vessel diameters in South African children.
3. To identify whether the associations of arterial modulation with physical fitness/activity, intake from food groups, and psychosocial behaviors are dependent on ethnicity.

### Study Design

**Figure 1:**
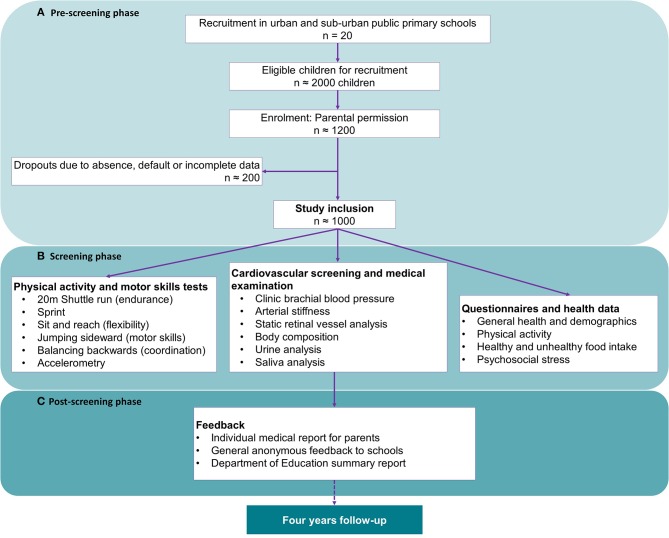
Flow diagram of the three phases of the baseline study population and data collection. **(A)** The pre-screening phase including the planning, organization, permission and recruitment; **(B)** The screening phase which involved participant assent and consent, and baseline data collection; and **(C)** The post-screening phase comprises of a multifaceted feedback process and data analysis.

The ExAMIN Youth SA study is an analytical, multidisciplinary, observational cohort study, designed to investigate the interplay between body composition, dietary intake, physical fitness, and physical activity, psychosocial stress, cardiovascular function as well as urinary and salivary biomarkers in 1,065 children (age 5–9 years) attending public primary schools in the North West province, South Africa. The study will obtain unique high-quality data at baseline and will expand the investigations after a 4-year follow-up period to identify exposures and early vascular changes that may contribute to cardiovascular disease development in children. An outline of the study design and procedures during the baseline phases are illustrated in Figure 1.

### Setting of the Study

**Figure 2:**
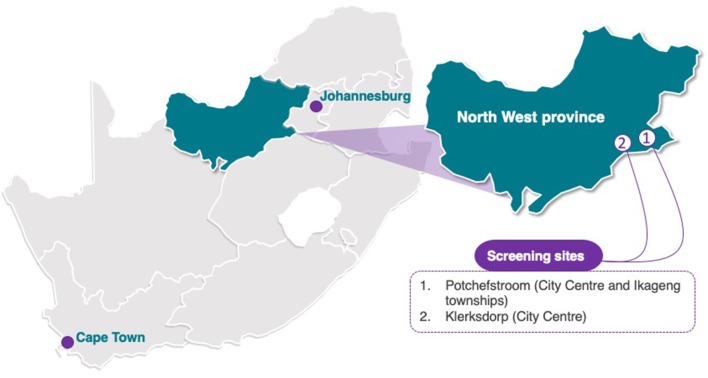
Data and sample collection for the ExAMIN Youth SA study took place in Potchefstroom and surrounding areas as well as in Klerksdorp, both located in the Southern district of the North-West province of South Africa. The outline of the South African map was purchased from yourfreetemplates.com with the Creative Commons' license, which is Attribution-NoDerivatives 4.0 International (CC BY-ND 4.0).

The study sites where data collection is performed are located within the Dr. Kenneth Kaunda district in two of the southern municipal areas namely JB Marks (Potchefstroom) and Matlosana (Klerksdorp) (Figure 2). The majority of the population in these areas consist of black (82%), with the remainder comprising of 14% white, 4% mixed-race, and 1% Indian, as reported on wazimap.co.za (accessed on 16 August 2019). All the data collection procedures are conducted in a school-based setting with the endorsement and support of the District Director's office of the Department of Education.

### Eligibility Criteria

Children between the ages of 5 and 9 years (both sexes and all ethnicities in the geographical region of investigation) are invited to participate voluntarily and with parental permission. No *exclusion criteria* are applied; however, children will be excluded if no informed consent were obtained.

### Recruitment and Informed Consent or Assent

The children are recruited from public schools, however heterogeneity in terms of socio-economic background is inevitable in the South African context. The Principal Investigator received endorsement and approval from the Department of Education to approach the school principals to present the project and discuss the details of each measurement and the purpose of the study. A school teacher, not directly involved with the particular children, will be identified as the mediator. Handout letters are circulated to the parents/caregivers to indicate their participation. Parents are invited to address any questions to the research team in order to make an informed decision and give parental permission for their child to participate in the study. All the relevant documentation including an informed consent/assent will then be provided to interested families. When the child along with the parents agree to participate, they will be requested to complete the informed consent/assent forms along with a battery of questionnaires prior to participation, and provide further consent/assent on the day of measurements.

## Data Collection

### Blood Pressure

To reduce inter-observer variability, validated (34, 35) and automated oscillometric pediatric blood pressure monitors [Omron HBP-1100-E, (OMRON Healthcare Co., Ltd. Kyoto, Japan)] will be used. The participants are requested to have avoided stimulant drugs or foods (self-reported), to sit quietly for 3–5 min with his or her back supported, feet on the floor and right arm supported, and the cubital fossa at heart level, before the blood pressure is measured (36, 37). The right arm is preferred in repeated measures of blood pressure for consistency and comparison to standard tables and because of the possibility of coarctation of the aorta, which might lead to false (low) readings in the left arm (38). Appropriately sized cuffs will be fitted for each child. Blood pressure measurements will be taken five times consecutively with 1-min resting intervals. The mean of the three measurements with the smallest variation will be used to calculate a mean for further analysis (22).

### Pulse Wave Analysis

Arterial pulse wave analysis will be performed using validated oscillometric Mobil-O-Graph monitors (I.E.M. GmbH, Germany) with integrated ARCSolver software (39, 40). The Mobil-O-Graph monitors are programmed to perform pulse wave analysis in duplicate after which the data will be downloaded for each participant using the HMS Client-Server software package Version 4.7.1 (I.E.M. GmbH, Germany). The participants will be fitted with an appropriately sized cuff for mid-upper right arm circumference in a sitting position. According to one measurement of brachial blood pressure, two measurements will be taken during the diastolic pressure for pulse wave analysis (pressure sensor MPX5050, Freescale Inc., Tempe, AZ, USA). This method determines parameters such as the central systolic blood pressure and central pulse pressure, the augmentation index and the standardized augmentation index at a heart rate of 75 beats/min, stroke volume, cardiac output, total vascular resistance, and aortic pulse wave velocity.

### Retinal Vessel Analysis

Static retinal blood vessel images will be captured using a Static Retinal Vessel Analyzer (SVA-T, Imedos Systems GmbH, Jena, Germany). The system consists of a fundus camera (Topcon TRC NW8) and analyzing software (Visualis 2.80, Imedos Systems GmbH, Jena Germany), allowing non-invasive and non-mydriatic assessment of retinal vessel diameters. Two valid images from the retina of both the left and right eye with an angle of 45° and with the optic disc in the center are taken per child. Retinal arterioles and venules, coursing through an area of 0.5–1 disc diameter from the optic disc margin, will semi-automatically be identified at higher magnification using the Vesselmap 2, Visualis, Imedos Systems GmbHsoftware. The examiner will differentiate all retinal arterioles and venules in the outer ring-zone and measure them with the software tools. Vessel diameters will be averaged to central retinal artery (CRAE) and vein equivalents (CRVE), using the Parr-Hubbard formula (41) and the arterio-venous ratio (AVR) will be subsequently determined (CRAE/CRVE). For the CRAE and CRVE we will use the mean of the right and the left eye results. Vessel diameters will be presented in measuring units (mu). In the model of Gullstrand's normal eye, 1 mu relates to 1 μm.

### Anthropometry and Bioelectrical Impedance Analysis

Anthropometric measures of height (cm) and weight (kg), waist circumference (cm), and skinfolds will be measured according to the International Society for the Advancement of Kinanthropometry (ISAK) procedures (42). Body mass is taken without shoes with the children dressed in light clothing with a Seca 813 digital scale to measure weight to the nearest 0.1 kg, and stature with a Seca 213 stadiometer (Birmingham, United Kingdom) with a perpendicular board to the nearest 1 mm. We will measure waist circumference consecutively in triplicate with a Lufkin® Executive thinline 2 mm steel tape (Apex Tool Group B.V.; AK Emmen, Netherlands) to the nearest 1 mm. Skinfolds of the triceps (measured parallel to the long axis of the arm at the triceps skinfolds site), and the subscapular (measured with the fold running obliquely downwards at the subscapular skinfold site) skinfolds, will be taken with a Harpenden skinfold caliper (Holtain Limited, U.K.) with a constant pressure of 10 g/mm^2^ to the nearest 0.2 mm. Skinfolds and waist circumference are carefully located using the correct anatomical landmarks. The sum of the triceps and subscapular skinfolds will be used to calculate the percentage body fat using the equations recommended for use in children from different settings (43).

Body composition will also be assessed using bioelectrical impedance analysis. The bioelectrical impedance analysis will be performed once using the Bodystat 1500® MDD (MultiScan 5000, BodyStat, Ltd). The bioelectrical impedance analysis detection electrodes are placed at the pisiform prominence of the right wrist and the anterior surface of the true ankle joint (44), which pass an imperceptible alternating current (frequency 50–60 hz) from one to another, measuring the impedance. A 200 g weight adjustment for clothing is applied. Measurements are conducted at a similar time of the day, after overnight fasting. Data from the bioelectrical impedance analysis include total body water, fat-free mass, lean body mass, body fat percentage, and total body fat in kilograms. Additionally, the bioelectrical impedance also provides data for impedance (Ω), resistance (Ω), and reactance (Ω).

### Physical Fitness

Prior to physical fitness tests, the children will perform a standardized 5-min warm-up. The test battery consists of a 20 m shuttle run (endurance), a 20-meter speed test (running speed), jumping sideward (coordination and agility), standing broad jump (strength), balancing backwards (balance), jumping, catching, kicking, and running (motor skill competences and coordination). All the tests will be performed during school time in the morning at the school with the same standardized equipment.

The following physical fitness tests are included:

1. The *20 m shuttle run* is a validated test measuring cardiorespiratory fitness by running back and forth in a 20 m distance (45). The initial running speed is set at 8.0 km/h with an increase of 0.5 km/h every minute, paced by beeps on an audio system. The maximal performance is achieved when the child did not cross the 20 m line at the moment of the beep for two consecutive 20 m distances. Numbers of “stages” achieved (1 stage ≈ 1 min) are counted with a precision of 0.5 stages (46). Participants are given a warning if they did not reach the line in time with the audio signal, and the test is ended if the participants could not reach the line for two successive shuttles or if the participant stopped voluntarily. But over and above this, the participants are verbally encouraged to perform maximally during each assessment and to run the test barefoot.
2. The *20 meter speed test* is measured using a 20-m sprint (47). Time is assessed by electronic timing gates of the Smart Speed testing device with a precision of 0.01s (HL2-31, Tag Heuer, La Chaux-de-Fonds, Switzerland). The start follows an acoustic signal. The faster of the two trials will be further analyzed.
3. Coordination or agility and muscular endurance are measured with the *jumping sideward* test (48). The children jump repeatedly, as many times as possible, within 15 s, with both legs together on alternating sides of a wooden strip. Five single test jumps will be performed per child prior to testing. This task is performed twice as fast as possible. The sum of the two trials are taken for the analysis.
4. Using a *balancing backwards* test on 3 m long bars with a starting width of 6 cm, followed by 4.5 and 3 cm, respectively, dynamic balance will be measured (48). Children are allowed to balance once forward and once backwards over the widest bar for familiarization. The number of steps until the child's foot touch the floor is counted. Three trials are performed for each bar width. The sum of these nine trials will be used for analyses.
5. The *standing broad jump* is designed to measure explosive strength (muscular fitness) and expressed in centimeters (cm) (49). Two trials will be given and the best trial recorded in centimeters. This test is performed on a non-slippery mat designed specifically for standing broad jump.
6. *Motor skills:* Competence in catching, kicking, running and jumping are assessed qualitatively and quantitatively. The Test of Gross Motor Coordination-2 (TGMD-2) (50) will be used to assess competence in each motor skill qualitatively. After demonstration by the tester, two attempts of each skill are performed and scored according to specific sub criteria for each skill (0 = no mastery, 1 = mastery), and then summed to obtain a competence score. A quantitative measure for each motor skill will also be determined. Catching and kicking accuracy are additionally scored out of 5 attempts, also using the TGMD-protocol (distances between testers and to the kicking target of 1.5 cm wide), while the distance jumped in the standing broad jump (49) and the time to complete the 20-meter speed test (47) will be used as quantitative measures for speed and jumping.

### Physical Activity Using Accelerometers

Physical activity will be further assessed using the ActiGraph GT3X (model 7164; Fort Walton Beach, FL, USA), which uses a solid-state triaxial accelerometer. The vertical axis is used as the cut-points and equations included for testing are determined based on accelerometer counts from the vertical axis. Before each experiment the accelerometer is initialized to collect data in 15-s epochs. Trained research assistants will fit the ActiGraphs on elasticized belts worn at the waist (just over the anterior superior iliac spine), according to manufacturer's instructions. The monitors are set to collect data in 30-s time increments for a time period of seven full days. Participants will be instructed to wear the waist-worn accelerometer 24-h/day for seven consecutive days. However, they are permitted to remove the accelerometers if they feel uncomfortable wearing the device during sleep. The participants will be advised to remove the accelerometers only during water-based activities such as bathing or swimming. Accelerometer data will be downloaded from the device to the computer using the ActiLife 6 Single (inc. 1 Full & 5 Lite activations) software. ActiGraph output data will be used as 15-s epochs or converted to 60-s epochs depending on the cut-point used. ActiGraph data will be classified as sedentary behavior (SB; <1.5 METs), light physical activity (LPA; 2.0 and ≤ 3.0 METs), or moderate to vigorous physical activity (MVPA; >3 METS) using ActiGraph cut-points.

### Questionnaires

#### General Health and Demographics Questionnaire

Socio-demographic information, including data on personal (age, sex, ethnicity) and family information (i.e., education of the parents, employment, type of dwelling, household amenities and marital status), as well as family history of cardiovascular disease, will be collected by means of a standard General Health Questionnaire. Parents are requested to assist in providing this information.

#### Physical Activity Questionnaire

A six-item questionnaire was developed and validated for completion by parents to provide information on their child (51). This questionnaire is self-administered, and will be sent home to the parents for completion and then sent back to the school on the day of study participation. The six questions included the usual amount of time per day spent in sleeping, naps, and various indoor and outdoor activities, which are grouped according to physical activity intensity (sedentary, moderate, and vigorous). From the information provided by parents through the questionnaire, the amount of daily time spent in different intensities of physical activity will be computed in minutes and as a percentage of time at home. Sports and supervised activities are recorded weekly and computed to minutes/day of different physical activity levels according to the reported activity. One additional question is asked to assess parents' overall perception of the child's typical physical activity level by assigning it to one of the three categories ranging from 1 (inactive) to 3 (very active).

#### Healthy and Unhealthy Food Intake Survey

We developed a simplified food frequency questionnaire to collect data on the consumption frequency of healthy and unhealthy food groups. The food groups included reflect foods reported to be eaten by South African school children and included four groups of healthy foods (fruits, vegetables, milk, meat/fish/poultry/eggs) and six groups of unhealthy foods (cold drinks, sweetened hot drinks, sweets, salty snacks, cakes and fast foods) (52, 53). This questionnaire is based on a questionnaire that was used in the WHO Global school-based student health survey (GSHS). The GSHS questionnaire concentrated on four food groups, fruits, vegetables, carbonated soft drinks and fast foods (54). Collection of reliable and accurate data on the dietary intake of young children is a challenge, therefore only intakes of different food groups were considered (55). A recent study showed that four dietary items of the School Physical Activity and nutrition questionnaire were sufficient to present a robust variable consistent with a healthy eating pattern (56). A similar questionnaire listing five healthy food groups (fruits, vegetables, legumes, fish, meat) and five unhealthy food groups (cold drinks, cookies, cake, candies, “ice pop”) with five different responses of frequency of intake was used to determine intakes of healthy and unhealthy foods of African children in Burkino Faso (57).

The face validity of the questionnaire has been assessed among Nutrition scientists with experience in dietary assessment. The scientists approved the questionnaire, but made recommendations about adding processed meat to the fast food group. This recommendation was implemented in the final questionnaire used in this study. The questionnaire was then pilot tested for comprehension in a group of caregivers of 6–8-year old children and was found to be easy to understand and complete. Healthy foods were defined as foods containing essential nutrients for child growth and general health, namely fruits, vegetables, milk, meat/fish/poultry/eggs (57). Unhealthy foods were defined as foods that provide energy, sugar, salt and fats, but do not make an important contribution to essential nutrient intake (54). A colored picture file with examples of foods from each group will be presented with the questionnaire to facilitate responses. This questionnaire is taken home to be completed with the assistance of the parent or caregiver and the child involved.

The median frequency of intake of each food group will be calculated, and categorized frequencies of food groups will be presented. Outcome data of participants with low frequencies of each food group (0–2 times/week) will be compared to data of participants with high frequencies (5–7 times/week) of each food group.

#### Psychosocial Behavior and Life Events

Parents will be requested to provide information about their own socio-economic background, their child's stress exposure and psychosomatic complaints via a questionnaire. Parents will complete two separate instruments to report their children's stress levels. To assess recent critical life events, parents/guardians should complete a 16-item adapted version of the *Life Events Checklist* (58). Parents should rate the impact of each event on their child's life, using a 4-point scale from 0 (no impact) to 3 (large negative impact). The mean influence of all events will be calculated as a trauma indicator. Thus, children with few life events with a strong influence could have higher scores compared to subjects with several but moderate life events. In addition, three sub-scales (family, friends, and school) of the KINDL^R^ questionnaire (59) will be used to assess specific sources of stress in the lives of the participating school children. Parents will be asked to respond on a 5-point Likert scale ranging from 0 (never) to 4 (all the time). The sub-scales used in this study were family (e.g., “my child got on well with us as parents,” “we quarreled at home”), friends (e.g., “my child got along well with his friends,” “my child felt different from other children”), and every day functioning at school (e.g., “my child easily coped with schoolwork,” “my child worried about his/her future”). Positively poled items will be recoded before computing the sub-scale mean scores, so that higher scores reflected increased psychosocial stress throughout all dimensions.

### Biological Sampling and Biochemical Analysis

#### Urine Sampling and Analysis

On the day before participation, a set of questionnaires, instructions for urine collection, all consumables for urine collection and information on the following day's measurements will be given to the child to take home after school. The following morning (day of participation), the participant is requested to collect a first voided morning midstream urine sample in the privacy of their own home with assistance from their parents. The filled urine container is then placed in the provided small cooling container with an ice pack. Urine samples are then collected at the schools and delivered to the laboratory for preparation according to standard procedures and stored in −80°C bio freezers prior to further analysis. Urinary biomarkers will be measured from stored urine samples including standard and novel biomarkers related to arterial modulation and nutrition. Initially, urinary albumin, creatinine, chloride, sodium, potassium, nitrite, nitrate, symmetric and asymmetric dimethylarginines, dimethylamine, malondialdehyde, and targeted metabolics will be measured.

#### Salivary Sampling and Analysis

On the day of participation in physical fitness assessments, each participant will be asked to provide a fasting saliva sample in provided SaliCaps (IBL International GmBH), before 8 am. Two additional saliva samples will be collected; one before the 20 m shuttle run test and another 30 min after the shuttle run intervention. This is done for a hypothalamic-pituitary adrenal axis reactivity intervention on cortisol levels, known to be involved in the harmful effects of stress exposures on cardiovascular function and biological aging (60). We will determine the salivary cortisol and estrone levels using solid phase enzyme-linked immunosorbent assays (ELISA) with the Synergy HT microplate reader, BioTek.

### Data Capturing, Management and Integrity

The ExAMIN Youth SA study will make use of the REDCap (Research Electronic Data Capture) system to capture all data elements. REDCap is a free, secure web-based, and user-friendly electronic database software which can be quickly developed and customized for studies for collecting and tracking information and data from research studies and scheduling patient visits (61). Each participant will receive a unique number linked to their data with no direct association to their personal information. A dedicated Data Manager ensures the correct handling of confidential information and provides anonymous and password protected datasets to the researchers using the data for analyses.

### Proposed Analysis

#### Sample Size Calculation

We aimed to include 20 urban schools from the Dr. Kenneth Kaunda district with a conservative calculation of 60 children participating per school, leading to a total sample size of ~1,200 children over a period of 19 months. However, with dropouts or school absentees we expected a total sample size of ~1,000 participants. Using G^*^Power version 3.1.9.2 (62), we determined the required effect size (*f* ^2^ = 0.025) for a given 2-sided level alpha error of probability at 0.05, in a total sample size of 1,065 and ten predictors for fixed model linear multiple regression analysis with blood pressure or pulse wave velocity or arteriolar-to-venular-ratio as main outcomes. With the proposed sample of 1,065 children, we achieve 95.3% power.

#### Statistical Analysis

The IBM^®^ SPSS^®^ Statistics software (Armonk, NY: IBM Corp.) will be used for data analysis. The 95% confidence intervals will be presented for measures of effect to indicate the amount of uncertainty. For future analysis, normality will be assessed by visual inspection using normal QQ plots. If residual plots indicate deviation from model norms, logarithmic transformations of the outcome variables will be considered. To describe continuous characteristics, we will use median and interquartile ranges or means and standard deviations; and for binary variables, we will use percentages by performing chi-square tests. To estimate absolute changes in outcome variables for one unit increase of determining factors, multiple linear regression analysis will be applied.

## Discussion

The ExAMIN Youth SA study is designed to contribute to the current lack of population level data on especially childhood hypertension and obesity by phenotyping target organ damage markers known to contribute to early vascular aging including arterial stiffness and retinal vessel diameters. In addition, we aim to investigate mediating risk factors such as physical inactivity, unhealthy dietary behavior and psychosocial stress.

It is evident from the literature that elevated blood pressure and large artery stiffness present itself at an earlier age in black than white populations (40–42). High blood pressure coincides with obesity, generating a vicious cycle that potentially fosters the development of early vascular changes in childhood. On a vascular level, elevated vascular resistance and large and small artery dysfunction are the driving forces for the development of cardiovascular disease and hypertension in particular (63, 64). Elevated blood pressure in childhood is the strongest predictor of adult hypertension. However, the global prevalence of childhood hypertension remains largely speculated and can range between 4 and 25% (3). Our study will contribute (although region specific) to the current lack of population level data in elevated blood pressure prevalence in South Africa by employing both European (65) and American (66) guidelines for the screening of high blood pressure in children. The role of physical activity, nutrition and psychosocial well-being in micro- and macrovascular health is still largely unexplored; and the ExAMIN Youth SA study will address these outcomes in order to elucidate the inevitable trend of early vascular aging in a bi-ethnic pediatric population.

The study does have some general limitations to report. The cohort will include volunteers as the children will not be randomly selected to participate. All children from the selected schools will be invited to participate and those with permission and consent from their parents and who gave assent will be included. The screening sites include only two (of three) municipality areas in one (of four) districts in the North West province of South Africa, making this cohort not representative of the whole province or the country. The accessibility to children younger than 5 years of age is extremely challenging and therefore we will include children between 5 and 9 years of age as these are the youngest ages at which we can observe potential signs of early vascular aging and potential health disparities among the groups to be studied in our cohort. With the ethical challenges to obtain blood samples from children, especially in a school-based setting, we will only obtain urine and saliva samples from the participants. With the stable nature of urine, we are able to measure a large number of metabolites and biomarkers which could aid in risk stratification and prospective risk prediction. The ExAMIN Youth SA study will generate new knowledge and contribute to the current limited data available, especially in an African context, on childhood health and in particular the interplay of physical inactivity, body composition, dietary behavior, and psychosocial determinants on micro- and macrovascular outcomes. The prospective nature of this observational cohort study will allow us to investigate potential identifiers of early cardiovascular risk in South African children by assessing target organ damage markers after 4-year of follow up and determine the predictors assessed in baseline. In addition, we have the opportunity to cross examine our main outcomes with a center in Basel, Switzerland with a similar cohort (22) of 6–8 year old children and identify similarities and differences of early vascular aging determinants between African and European children.

In conclusion, the ExAMIN Youth SA study will assess the prevalence of two important global childhood conditions namely obesity and hypertension while considering ethnicity. This study will also focus on mediators in large artery stiffness and retinal microvascular diameters to understand the cross-talk between the vascular beds. Additional analysis of biomarkers will aid our understanding of the molecular determinants of early cardiovascular compromise in children and development of manifest cardiovascular disease later in life.

## Ethics Statement

The studies involving human participants were reviewed and approved by Health Research Ethics Committee of the North-West University. Written informed consent to participate in this study was provided by the participants' legal guardian/next of kin.

## Author Contributions

RK is the Principal Investigator of the ExAMIN Youth SA study, responsible for the design and execution of the study, drafting of the protocol, securing funding, and ethics monitoring of the study progress. MM is a senior co-investigator of the study and plays a major role in participant recruitment and stakeholder reporting, data collection, data entry, and drafting of this paper. AS contributed to the scientific integrity of the study and gave input in the drafting of this paper. WS is our expert on the retinal vessel analysis and contributes to data collection and drafting of this paper. CM is our biochemistry expert and the laboratory manager handling all the biological samples and planning of future analyses. HK is the nutrition expert on the team and developed the South African specific healthy and unhealthy food intake questionnaire, conducted the face validity thereof, data collection, and assisted in drafting this paper. AP is the expert on physical fitness and physical activity in children, assists with data collection, and drafting of this paper. LG-M assists with blood pressure data collection, data cleaning, and drafting of this paper. YB assists with static retinal vessel analysis data collection and drafting of this paper. LL assists with pulse wave analysis data collection, data cleaning, and drafting of this paper. GM assists with static retinal vessel analysis data collection, saliva sampling, and drafting of this paper. AK and ED assist with the development and translation of the psychosocial questionnaires, data collection, and drafting of this paper. HH conceptualized and is the Principal Investigator of the ExAMIN Youth study in Basel, Switzerland, supports image acquisition of retinal vessels and is responsible for retinal vessel analysis, international collaborator of the ExAMIN Youth SA study, critically revised the manuscript. All authors read and approved the final manuscript.

## Conflict of Interest

The authors declare that the research was conducted in the absence of any commercial or financial relationships that could be construed as a potential conflict of interest.

## Abbreviations

ABET: adult-based education and training
AVR: arterio-venous ratio
CRAE: central retinal artery equivalent
CRVE: central retinal vein equivalent
ELISA: enzyme-linked immunosorbent assay
ExAMIN, exercise: arterial modulation and nutrition
KINDL^R^: revidierter KINDer scientific research-academic medical center child quality-of-life questionnaire
REDCap: research electronic data capture
TGMD-2: test of gross motor development.
